# Comparison of sexual function, urinary symptoms, bowel function, postoperative pain, and cosmetic satisfaction after vaginal natural orifice transluminal endoscopic surgery and total laparoscopic hysterectomy

**DOI:** 10.1590/1806-9282.20252179

**Published:** 2026-07-24

**Authors:** Mehmet Emre Peker, Ufuk Atlıhan, Duygu Uçar Kartal, Can Ata, Onur Yavuz, Aygül Aliyeva

**Affiliations:** 1 Manisa Merkezefendi State Hospital – Manisa, Turkey.; 2 Izmir Buca Seyfi Demirsoy Training and Research Hospital – Izmir, Turkey.; 3 Dokuz Eylül University Hospital – Izmir, Turkey.; 4 Private Memorial Hospital – Istanbul, Turkey.

**Keywords:** Natural Orifice Endoscopic Surgery, Laparoscopy, Hysterectomy, Sexual health, Patient satisfaction

## Abstract

**OBJECTIVE:**

The aim of this study was to compare postoperative sexual, urinary, and bowel function, pain, and cosmetic satisfaction in women undergoing hysterectomy via vaginal natural orifice transluminal endoscopic surgery or total laparoscopic hysterectomy for benign indications.

**METHODS:**

This retrospective study evaluated 43 patients who underwent total laparoscopic hysterectomy (n=25) or vaginal natural orifice transluminal endoscopic surgery (n=18) between January 2021 and June 2025. Functional outcomes were assessed using Female Sexual Function Index, Urogenital Distress Inventory-6, and Wexner scores; pain and cosmetic satisfaction were measured with Visual Analog Scale. Appropriate statistical tests were applied with p<0.05 considered significant.

**RESULTS:**

Sexual, urinary, and bowel outcomes were similar between groups (p>0.05). Pain scores at 24 and 48 h were significantly lower in the vaginal natural orifice transluminal endoscopic surgery group (p=0.012 and p=0.028), and cosmetic satisfaction was higher (p=0.004).

**CONCLUSION:**

Vaginal natural orifice transluminal endoscopic surgery and total laparoscopic hysterectomy yield comparable functional outcomes, but vaginal natural orifice transluminal endoscopic surgery demonstrates advantages in postoperative pain and cosmetic satisfaction.

## INTRODUCTION

Hysterectomy is among the most frequently performed gynecologic operations and is preferred for the treatment of various benign or malignant conditions in many women^1^. With advances in surgical techniques, minimally invasive approaches have gained popularity and offer advantages over open surgery, including faster recovery, reduced pain, and shorter hospital stays^2^. Total laparoscopic hysterectomy (TLH) is widely used and considered both safe and effective^3^. More recently, vaginal natural orifice transluminal endoscopic surgery (vNOTES) has emerged as an appealing alternative, combining the benefits of laparoscopy with the natural transvaginal route^4^.

The vNOTES technique avoids abdominal incisions and may improve cosmetic outcomes, postoperative pain, and recovery time^5^. Current evidence suggests that vNOTES is safe, with operative time and blood loss comparable to or lower than TLH^6^. However, as a relatively new technique, long-term outcomes remain limited, and further research is needed to assess effects on sexual function, urinary symptoms, pelvic floor health, and bowel habits^7^.

Sexual function is a key component of well-being and may change after hysterectomy^8^. Factors such as surgical technique, tissue manipulation, postoperative pain, and recovery duration may influence outcomes. While TLH has been well studied, results remain heterogeneous^9^. Early findings for vNOTES suggest faster recovery and earlier return to sexual activity, but larger studies are needed to confirm these results^10^.

Urinary symptoms and pelvic floor function are also important considerations. Preservation of pelvic support structures and intraoperative manipulation may affect postoperative incontinence, voiding function, and overactive bladder symptoms^11^. Because vNOTES aims to minimize pelvic floor trauma, its potential advantages in urinary outcomes are of particular interest. Similarly, postoperative bowel symptoms such as constipation or discomfort may vary between approaches, although current evidence is inconclusive^12^.

Postoperative pain and cosmetic satisfaction are central endpoints in minimally invasive surgery. The absence of abdominal incisions in vNOTES may reduce pain, analgesic use, and scarring, whereas TLH requires multiple abdominal trocar sites^5,6^. Evaluating these outcomes from a patient-centered perspective is essential.

Although previous studies have compared vNOTES and TLH, most have focused on intraoperative or early postoperative parameters such as operative time, bleeding, and complications^1,4,7^. Patient-centered outcomes—including sexual, urinary, and bowel function, postoperative pain, and cosmetic satisfaction—remain equally important.

Therefore, this study aimed to compare postoperative sexual function, urinary and bowel function, pain levels, and cosmetic satisfaction between patients undergoing vNOTES and TLH. These findings may support more informed clinical decision-making and guide future research in minimally invasive gynecologic surgery.

## METHODS

This single center retrospective comparative study was conducted at the Department of Obstetrics and Gynecology of Buca Seyfi Demirsoy Training and Research Hospital. Ethical approval for the study was obtained from the institutional ethics committee (Approval No: 2026/2-20), and the research was carried out in accordance with the principles of the Declaration of Helsinki. Patients who underwent TLH or vNOTES for benign gynecologic indications between January 2021 and June 2024 were identified through operating room records, electronic medical files, and outpatient follow-up notes. Women aged 18–70 years with at least 12 months of postoperative follow-up were included. The median duration of postoperative follow-up was 24 months (range: 12–42). Patients were excluded if they had suspected or confirmed malignancy, underwent any concomitant pelvic floor or reconstructive surgery (including procedures that could affect urinary, bowel, or sexual function), had advanced-stage endometriosis, had missing data in functional assessment scales, or required reoperation due to postoperative complications. Based on these criteria, a total of 43 patients were evaluated, including 25 in the TLH group and 18 in the vNOTES group.

All surgical procedures were performed by the same experienced gynecologic laparoscopic surgical team. In the TLH group, a standard four-trocar laparoscopic approach was used; the uterine vessels were coagulated with bipolar energy, the uterus was removed vaginally, and the vaginal cuff was closed laparoscopically. In the vNOTES group, a transvaginal access platform consisting of an Alexis wound retractor (Applied Medical, Rancho Santa Margarita, CA, USA) combined with a surgical glove port was inserted following anterior or posterior colpotomy, and all surgical steps were performed using transvaginal endoscopic techniques. The vaginal cuff was closed transvaginally. Intraoperative and postoperative complications were recorded and classified according to the Clavien-Dindo classification system. Intraoperative complications included events such as organ injury, excessive bleeding requiring intervention, or conversion to laparotomy. Postoperative complications occurring within 30 days after surgery included vaginal cuff infection, pelvic hematoma, urinary tract infection, and other procedure-related adverse events. Demographic and clinical characteristics, intraoperative and early postoperative outcomes, and functional evaluation data were systematically recorded. Variables such as age, body mass index, parity, menopausal status, history of vaginal delivery or cesarean section, indications for hysterectomy, and complication rates were summarized in tables. Ovarian conservation or oophorectomy was performed according to patient age, menopausal status, and clinical indication. No routine prophylactic oophorectomy was performed in premenopausal patients.

The choice between vNOTES and TLH was determined based on a combination of surgeon experience, patient preference, and individual clinical characteristics. Factors influencing the decision included uterine size, vaginal accessibility, history of previous abdominal or pelvic surgery, and pelvic anatomy. vNOTES was generally preferred in patients with favorable vaginal access, absence of suspected severe adhesions, and uterus size suitable for transvaginal removal. Conversely, TLH was selected in cases with limited vaginal access, suspected adhesions, or when transvaginal access was considered technically challenging. All patients were informed about both surgical options, and the final decision was made through shared decision-making between the surgeon and the patient.

Sexual function was assessed using the Female Sexual Function Index (FSFI), urinary symptoms using the Urogenital Distress Inventory-6 (UDI-6), and bowel function using the Wexner Constipation Score. Postoperative pain at 24 and 48 h was evaluated using the Visual Analog Scale (VAS), and cosmetic satisfaction was assessed using a categorical patient-reported outcome indicating “very satisfied.” Validated Turkish versions of these instruments were used, and their reliability and validity in the Turkish population have been previously demonstrated. In this retrospective study, questionnaire data were obtained from outpatient follow-up records. When necessary, patients were contacted by telephone to complete missing questionnaire data. Statistical analyses were performed using Statistical Package for the Social Sciences (SPSS) version 27.0 (IBM Corp., Armonk, NY, USA). An a priori sample size calculation was performed using G*Power 3.1, which indicated that at least 17 patients were required in each group (total n=34) to detect a moderate effect size (d=0.7) in postoperative VAS pain scores with a twotailed α=0.05 and 80% power. Since continuous variables did not show normal distribution, the Mann-Whitney U test was used for comparisons, while categorical variables were analyzed using the chi-square test or Fisher’s exact test when appropriate. A p-value of <0.05 was considered statistically significant.

## RESULTS

A total of 43 patients were included in the study, of whom 25 underwent TLH and 18 underwent the vNOTES approach. The median follow-up duration was 24 months (range: 12–42), with no significant difference between the groups (p>0.05). The demographic and clinical characteristics of the patients were similar between the two groups. There were no significant differences in age, body mass index, or parity (p>0.05). The proportion of postmenopausal patients was 64.0% in the TLH group and 55.6% in the vNOTES group, with no statistically significant difference between the groups (p=0.760). Likewise, the distribution of vaginal delivery history, previous cesarean sections, and indications for hysterectomy did not differ significantly between the groups (p>0.05). The most common surgical indication in both groups was symptomatic fibroids (48.0% in TLH vs. 44.4% in vNOTES).

The operative time was 63 min (45–75) in the TLH group and 61 min (50–70) in the vNOTES group, with no significant difference between the two techniques (p=0.79). Hematocrit drop and length of hospital stay were also similar between the groups (p=0.62 and p=0.84, respectively). The intraoperative complication rate was 8.0% in the TLH group and 5.6% in the vNOTES group. In the TLH group, one patient experienced minor bleeding requiring additional bipolar coagulation, while in the vNOTES group one patient had a limited vaginal wall laceration that was repaired intraoperatively. The 30-day postoperative complication rates were 8.0 and 11.1%, respectively, including cases of urinary tract infection and minor vaginal cuff infection. All complications were managed conservatively with appropriate medical treatment, and none required reoperation or conversion to laparotomy. No statistically significant difference was observed between the groups (p=1.000) (Table 1).

**Table 1:** Intraoperative and early postoperative outcomes.

Parameter	TLH (n=25)	vNOTES (n=18)	p-value
Operative time (min), median (IQR)	63 (45–75)	61 (50–70)	0.79
∆ Hematocrit (%), median (IQR)	3.0 (1.9–4.7)	2.6 (1.4–4.1)	0.62
Length of hospital stay (days), median (IQR)	1.4 (1–4)	1.6 (1–4)	0.84
Intraoperative complications, n (%)	2 (8.0%)	1 (5.6%)	0.82
Postoperative complications (≤30 days), n (%)	2 (8.0%)	2 (11.1%)	0.96
Conversion to laparotomy, n (%)	0 (0.0%)	0 (0.0%)	—

TLH: total laparoscopic hysterectomy; vNOTES: vaginal natural orifice transluminal endoscopic surgery; IQR: interquartile range.

There were no significant differences between the two groups in terms of sexual function, urinary symptoms, or bowel function (Table 2). The preoperative total FSFI scores were 26.4 in the TLH group and 26.2 in the vNOTES group (p=0.88). Although a slight decline in FSFI scores was observed 6–12 months postoperatively in both groups, the magnitude of change did not differ significantly (-1.6 in TLH vs. -1.1 in vNOTES; p=0.69). UDI-6 scores were similar in both the preoperative and postoperative periods; postoperative scores were 12 in the TLH group and 10 in the vNOTES group, with no statistically significant difference (p=0.33). Likewise, changes in Wexner constipation scores from preoperative to postoperative assessments did not differ between the groups (p>0.05).

**Table 2:** Sexual function, urinary distress, and bowel function scores.

Parameter	TLH (n=25)	vNOTES (n=18)	p-value
FSFI total score, preoperative	26.4 (23.6–28.9)	26.2 (23.2–29.3)	0.88
FSFI total score, 6–12 months postop	24.8 (21.9–27.6)	25.1 (22.4–28.1)	0.74
∆ FSFI (postop–preop)	-1.6	-1.1	0.69
UDI-6 score, preoperative	15 (10–18)	14 (9–17)	0.81
UDI-6 score, postoperative	12 (8–15)	10 (6–12)	0.33
∆ UDI-6 (postop–preop)	-3	-4	0.41
Wexner constipation score, preoperative	6.3 (4–9)	6.5 (4–8)	0.90
Wexner constipation score, postoperative	5.1 (3–8)	4.4 (2–7)	0.56
∆ Wexner (postop–preop)	-1.2	-2.1	0.48

FSFI: Female Sexual Function Index; UDI-6: Urogenital Distress Inventory-6; TLH: total laparoscopic hysterectomy; vNOTES: vaginal natural orifice transluminal endoscopic surgery.

When postoperative pain and cosmetic satisfaction were evaluated, the vNOTES technique demonstrated a clear advantage (Table 3). The 24-h postoperative VAS pain score was significantly lower in the vNOTES group compared to the TLH group (4.2 vs. 5.6; p=0.012). Similarly, the 48-h pain scores were also lower in favor of vNOTES (3.0 vs. 3.9; p=0.028). The proportion of patients reporting being “very satisfied” with their cosmetic outcome was significantly higher in the vNOTES group (83.3%) compared with the TLH group (40.0%) (p=0.003).

**Table 3:** Postoperative pain, analgesic requirements, and cosmetic satisfaction.

Parameter	TLH (n=25)	vNOTES (n=18)	p-value
VAS pain score at 24 h (0–10), median (IQR)	5.6 (4.6–7.1)	4.2 (3.2–5.3)	0.012
VAS pain score at 48 h (0–10), median (IQR)	3.9 (2.8–4.9)	3.0 (2.2–4.1)	0.028
“Very satisfied” with cosmetic result, n (%)	10 (40.0%)	15 (83.3%)	0.003

VAS, visual analog scale; TLH: total laparoscopic hysterectomy; vNOTES: vaginal natural orifice transluminal endoscopic surgery; IQR: interquartile range.

## DISCUSSION

In this retrospective study, vNOTES and TLH were compared in patients undergoing hysterectomy for benign gynecologic indications in terms of sexual function, urinary symptoms, bowel function, postoperative pain, and cosmetic satisfaction. Our findings indicate that both techniques yield similar functional outcomes, whereas vNOTES provides a significant advantage regarding postoperative pain and cosmetic satisfaction. These results are consistent with previous studies reporting the growing role of vNOTES among minimally invasive surgical options^3,4,5^.

The similarity in demographic characteristics and surgical indications between the two groups enhances the interpretability of the findings, independent of patient selection. Although Wattiez and colleagues^2^ suggested that the learning curve of laparoscopic surgery may influence outcomes, the fact that all procedures in our study were performed by an experienced surgical team minimizes this potential confounder. Furthermore, Donnez and colleagues^3^ reported that minimally invasive approaches provide comparable clinical outcomes in appropriately selected cases, which aligns with our observations.

The comparable operative times between the groups are in agreement with the results of Chen et al.^9^ and Perron-Burdick et al.^10^, demonstrating that vNOTES does not prolong surgical duration. The similarity in hematocrit decline and hospital stay is also consistent with the safety profile reported by Uccella et al.^11^ in their series of laparoscopic hysterectomies.

With regard to sexual function outcomes, the slight postoperative decline in FSFI scores appears to be independent of surgical technique. Thakar et al.^12^ reported that sexual function remains stable in most women after hysterectomy, while Roovers et al.^13^ similarly demonstrated no major changes in postoperative sexual function. The emphasis by von Schoultz et al.^14^ on the hormonal and psychological influences in this context further supports our conclusion that surgical technique is not a primary determinant. Contrary to Chai et al.^15^, who suggested that vNOTES may facilitate earlier recovery of sexual activity, our study found no clinically meaningful difference between the groups. Although the present study found no significant difference in postoperative FSFI scores between the vNOTES and TLH groups, sexual function is a complex outcome influenced by multiple biopsychosocial factors beyond surgical technique. Psychological well-being, relationship dynamics, and hormonal status may play a substantial role in postoperative sexual outcomes. In this context, Lerner et al. demonstrated that cognitive-behavioral therapy significantly improved sexual desire and overall sexual function in women with hypoactive sexual desire disorder, highlighting the critical role of psychological factors in female sexual health^16^. Therefore, the absence of a significant difference between surgical approaches may reflect the multifactorial nature of sexual function rather than a true equivalence of surgical impact alone. Future studies evaluating sexual function after hysterectomy should consider incorporating multidisciplinary and patient-centered approaches.

Regarding urinary symptoms, the absence of a significant difference between vNOTES and TLH aligns with the findings of Erekson et al.^17^ and Barber^18^, who reported that postoperative urinary function changes are largely related to pelvic support structures. Although Baekelandt et al.^19^ suggested that vNOTES may be less traumatic to the pelvic floor, our findings indicate no long-term functional advantage.

Similarly, the comparable changes in Wexner constipation scores between the groups are in accordance with the studies by Altman et al.^20^ and Gustafsson et al.^21^, which found that hysterectomy generally has limited effects on bowel function. Therefore, our results suggest that vNOTES does not confer additional benefit in this domain.

Postoperative pain outcomes clearly favored vNOTES, in agreement with the findings of Baekelandt et al.^5^, who reported reduced postoperative pain with vNOTES hysterectomy. Wong et al.^22^ also emphasized that the absence of abdominal incisions in natural orifice surgery contributes to reduced pain levels. Additionally, the significantly higher cosmetic satisfaction observed in the vNOTES group is supported by the findings of Li et al.^23^, who showed that vNOTES techniques offer superior aesthetic outcomes compared with laparoscopic approaches. However, cosmetic satisfaction was evaluated using a simple patient-reported categorical measure rather than a validated scoring system, which should be considered when interpreting these results. Recent studies have also highlighted the expanding role of vNOTES in pelvic reconstructive procedures, demonstrating favorable surgical and functional outcomes in the management of apical prolapse^24^.

In addition to hysterectomy and pelvic reconstructive procedures, vNOTES has increasingly been utilized in a variety of gynecologic indications. Recent studies have demonstrated its feasibility and safety in adnexal surgeries, including ovarian cystectomy and salpingo-oophorectomy, as well as in the management of ectopic pregnancy. Furthermore, vNOTES has been reported as a promising approach in selected cases of early-stage gynecologic malignancies and staging procedures, highlighting its expanding role in minimally invasive gynecologic surgery^25,26,27^.

The main limitations of this study include its retrospective design, relatively small sample size, and the potential variability in outcomes based on surgical expertise. In addition, although the sample size calculation was based on postoperative pain scores, the relatively small number of patients may have limited the statistical power to detect differences in secondary outcomes such as sexual function, urinary symptoms, and bowel function. Therefore, the possibility of a type II error should be considered when interpreting these results. Since the surgical approach was not randomized, a certain degree of selection bias cannot be excluded. However, performing all surgeries by the same experienced team reduces this limitation. Moreover, the inclusion of at least 12 months of follow-up provides a more reliable assessment of functional outcomes.

In conclusion, vNOTES and TLH are both safe and effective surgical options for benign gynecologic indications. Although functional outcomes did not differ significantly between the groups, vNOTES demonstrated notable advantages in postoperative pain and cosmetic satisfaction. vNOTES appears to be a valuable surgical option that may enhance patient satisfaction when appropriately selected. Larger prospective and randomized studies are essential to further validate these findings and guide clinical practice.

## Data Availability

The datasets generated and/or analyzed during the current study are available from the corresponding author upon reasonable request.
